# Supplementary material for: Causal interplay between lactose intolerance and gut microbiota: a combined bidirectional Mendelian randomization and in vivo validation study
Source: Front Nutr. 2026 Jun 1;13:1803337. doi: 10.3389/fnut.2026.1803337 (PMC13265576; doi:10.3389/fnut.2026.1803337)
Supplement: Supplementary file 1 [file Data_Sheet_1.zip › supplementary materials/Forward/leave one out cross validation/ebi-a-GCST90027455.finngen_R12_E4_LACTONAS.pdf]

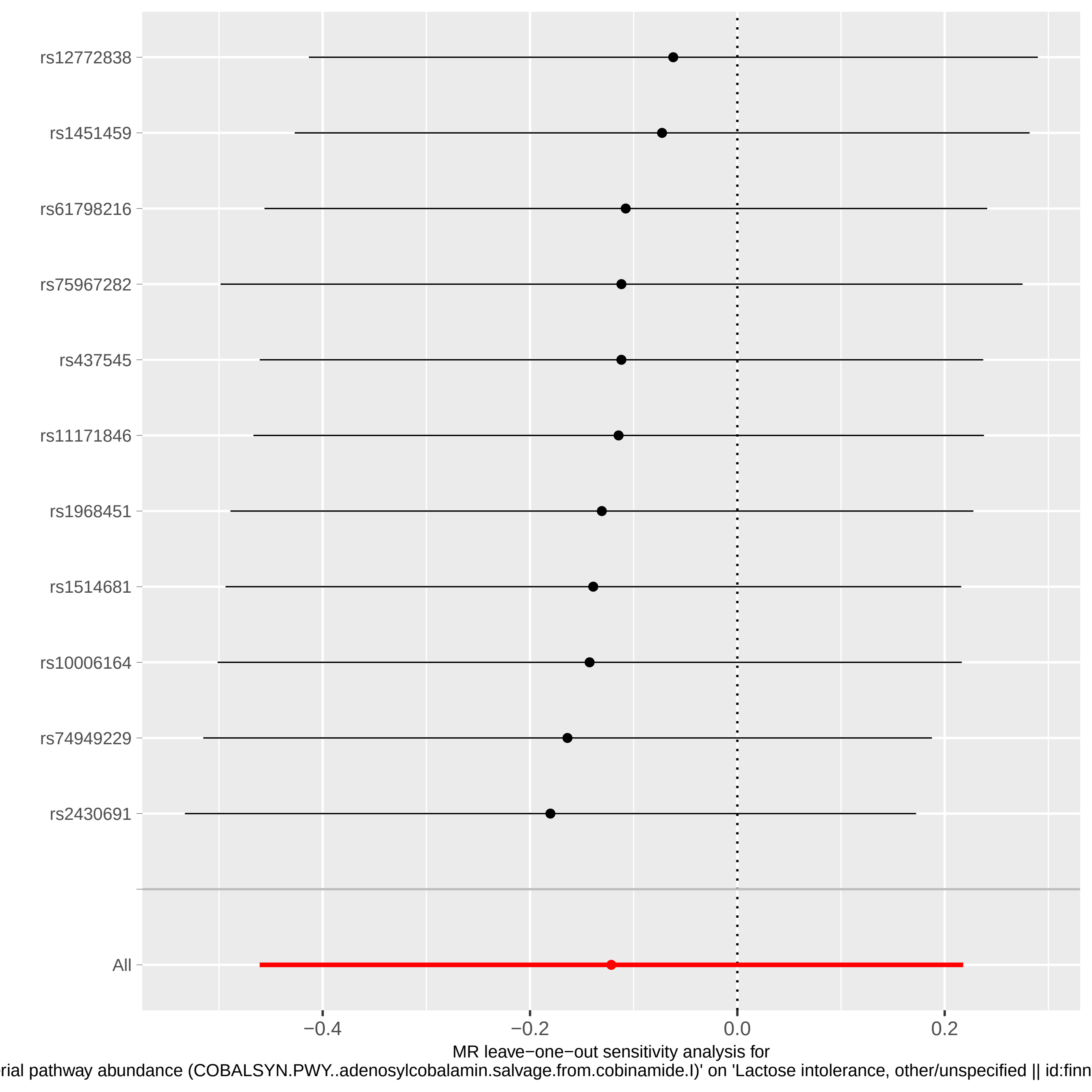
rs12772838
rs1451459
rs61798216
rs75967282
rs437545
rs11171846
rs1968451
rs1514681
rs10006164
rs74949229
rs2430691
All
−0.4
−0.2
0.0
0.2
MR leave−one−out sensitivity analysis for
erial pathway abundance (COBALSYN.PWY..adenosylcobalamin.salvage.from.cobinamide.I)' on 'Lactose intolerance, other/unspecified || id:finn
